# Destructive effect of HIFU on rabbit embedded endometrial carcinoma tissues and their vascularities

**DOI:** 10.18632/oncotarget.14751

**Published:** 2017-01-19

**Authors:** Liming Guan, Gang Xu

**Affiliations:** ^1^ Department of Obstetrics and Gynaecology, Zhabei District Central Hospital, Zhabei District, Shanghai 200000, China; ^2^ Department of Radiotherapy, Tumor Hospital, Peking University, Fengtai District, Beijing 100000, China

**Keywords:** high intensity focused ultrasound(HIFU), tumor vascularities, endometrial cancer(EC)

## Abstract

**Objectives:**

To evaluate damage effect of High-intensity focused ultrasound on early stage endometrial cancer tissues and their vascularities.

**Materials and Methods:**

Rabbit endometrial cancer models were established via tumor blocks implantation for a prospective control study. Ultrasonic ablation efficacy was evaluated by pathologic and imaging changes. The target lesions of experimental rabbits before and after ultrasonic ablation were observed after autopsy. The slides were used for hematoxylin-eosin staining, elastic fiber staining and endothelial cell staining; the slides were observed by optical microscopy. One slide was observed by electron microscopy. Then the target lesions of experimental animals with ultrasonic ablation were observed by vascular imaging, one group was visualized by digital subtract angiography, one group was quantified by color Doppler flow imaging, and one group was detected by dye perfusion.

SPSS 19.0 software was used for statistical analyses.

**Results:**

Histological examination indicated that High-intensity focused ultrasound caused the tumor tissues and their vascularities coagulative necrosis. Tumor vascular structure components including elastic fiber, endothelial cells all were destroyed by ultrasonic ablation. Digital subtract angiography showed tumor vascular shadow were dismissed after ultrasonic ablation. After ultrasonic ablation, gray-scale of tumor nodules enhanced in ultrasonography, tumor peripheral and internal blood flow signals disappeared or significantly reduced in color Doppler flow imaging. Vascular perfusion performed after ultrasonic ablation, tumor vessels could not filled by dye liquid.

**Conclusion:**

High-intensity focused ultrasound as a noninvasive method can destroy whole endometrial cancer cells and their supplying vascularities, which maybe an alternative approach of targeted therapy and new antiangiogenic strategy for endometrial cancer.

## INTRODUCTION

### Personal treatment of endometrial cancer

Endometrial cancer (EC) is one of gynecologic malignant tumor. The occurrence rate of endometrial cancer has been increasing in recent years. It was presumed 46,470 women become new patients and 8,120 women die from Endometrial cancer in United States. Contrast to the incidence of many tumor types are decreasing, the occurrence rate of EC has been increasing in former 30 years around the world. General early stage endometrial cancer can be cured. While advanced stage endometrial cancer and relapsing endometrial cancer may not easily be treated [1].

According to tumor classification of endometrial cancer in 2009, tumor within uterus no outside of uterine serosa invasion was all considered as stage I. Clinical observation showed that lymph node dissection in early stages of endometrial cancer could not increase patients survival rate, so this process can be deleted [2].

Endometrial cancer treatment methods mainly include surgery, radiation, and chemotherapy. Effect of these therapy is limited to early stage endometrial tumor. Chemotherapy is associated with toxic effect such as ovarian failure. As growing knowledge of tumor molecular mechanism, molecular elements of tumor may be used as targets, this method is called target therapy. Molecular pathways are often correlative and unstable, so it was complicated process for us to select qualified agents and obtain a satisfying clinical trial effect [1, 2].

### Targeting of tumor vascular treatment

Tumor blood vessels can offer energy and nutriments for tumor. Chaotic vasculogenesis induces tumor abnormal metabolism, growth and invasion. Antiangiogenesis therapy has enjoyed equal popularity just like chemical and radiation therapy in antitumor treatment. Now only a small part of patients with cancer can be cured by anti-angiogenic agents. Unstable and varied conditions of tumor are easy cause resist to the treatment. While side effects of the drugs make investigators look for more effective agents [3].

Effects of antiangiogenic agents such as avastin were dissatisfied [4], as an alternative approach of targeted therapy, high intensity focused ultrasound(HIFU) may used in this fields.

### Uterine myoma treated by HIFU

As an acoustic energy, high intensity ultrasound can be used to ablate tumor tissue precisely through heat effect and cavitation effect. Hysteromyomas is the most common gynecologic benign tumors, As a non-invasive method, it is confirmed HIFU can ablate hysteromyomas with satisfied effects, few treatment complications [5].

HIFU provides an excellent approach to ablate hysteromyomas, but no studies are available in the literature related to malignant gynecological tumors such as endometrial cancer ablated with HIFU.

In this study, tumor blocks were implanted into uteruses of experimental rabbits, impact by HIFU on early stage endometrial carcinoma tissues(tumor nodule within myometrium) and their microvessels were observed by pathologic and imaging methods.

## RESULTS

### Uterine carcinoma microvascular construction versus HIFU treatment

#### Hematoxylin-eosin detection

**Figure 1:**
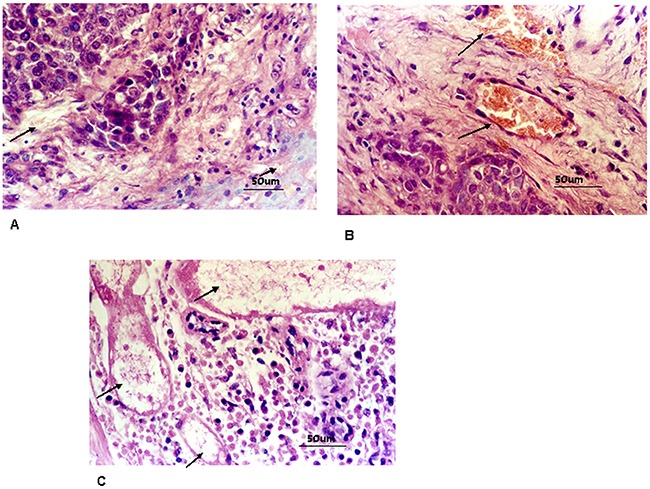
**A**. Control group: blood capillary inside uterine tumor nest were integrated (marked with arrows,400 times HPF). **B**. Control group:blood capillary and venule peripheral to uterine tumor nest were integrated (marked with arrows,400 times HPF). **C**. In HIFU treated group, cell shrinkage, nucleon deeply stained, loose cell intervals. Thrombi were found within target vessels. (marked with arrows, HE staining, 400 times HPF).

Uterine tumor microvessels included capillaries, small veins as well as small arteries. Tumor vascular structures were intact internal or surrounding tumor nodules in control group(Figure 1A, Figure 1B).

The mean number of uterine carcinoma microvessels altogether in control group was 10.23 ± 2.35/vessels/HPF (high power field,400 times).

By autopsy of general observation in HIFU treated group, the treated range no hemorrhage occurred, this result validated HIFU treatment was secure. General examination showed that HIFU treatment induced the ablated region become necrosed tissues completely. The scope included uterine tumors and a mean border wide 1.86±0.37cm (range:1.7 to 2.1cm) of normal uterine tissues around tumor nodules. The tissues within the treated scope were all degenerative necrosis. It is not found living tumor cells left at the centre of uterine tumor and at border of uterine tumor in HIFU ablated group. The treatment induced the uterine tissue rigid and there was distinct border in the middle of the ablated and no ablated uterine tissue.

In HIFU treated group, tumor appeared cell shrinkage, nucleon deeply stained, loose cell intervals. Cellular outlines were still distinct. Vascular composition was completely damaged. Vascular cellular intervals were indistinct, endothelium collapsed, cell nucleus disappeared, media vascular were disrupted. Thrombi were found within target vessels, Tumor vessels all become necrosed tissues. Necrotic tissue decomposition presented at 24 h after HIFU ablation. (Figure 1C).

#### Vascular elastan detection

**Figure 2:**
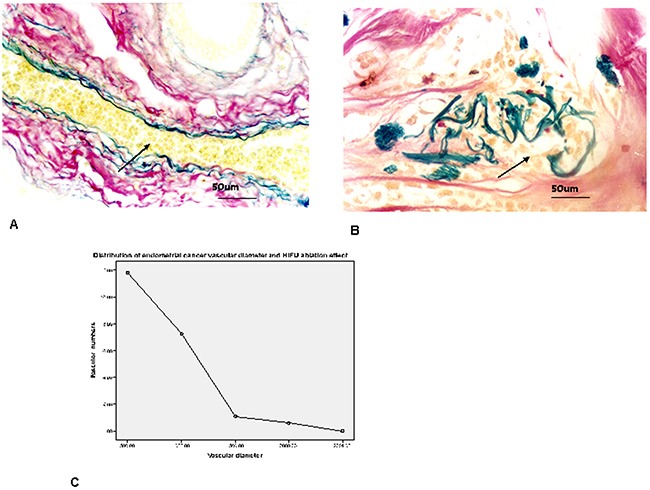
**A**. Tumor vascular internal and external elastic plates were intact (elastic fiber marked with arrows, elastic and collagen fibers staining, elastic fibers presented blue, collagen fibers appeared red and background presented a little yellow, vascular caliber 60 μm, 400 times HPF). **B**. The vascular elastic fiber plates were decomposed. Elastic fiber arrangement was in chaos status, fragmented or layered distribution in HIFU treated group (elastic fiber marked with arrows, elastic and collagen fibers staining, vascular caliber 100 μm, 400 times HPF). **C**. The calibers of uterine tumor microvessels were all within 2000μm in both groups. most of vascular caliber within 500μm; smaller vascular caliber was associated with better ablated effect by HIFU.

Vascular elastan presented blue, vascular collagen appeared red and surroundings presented a little yellow. The inside and outside elastan layers of the uterine tumor vasculature were integrated in control group(Figure 2A). The vascular elastan plates were decomposed in HIFU ablated group. Elastan layer arrangement was in chaos status, fragmented or layered distribution (Figure 2B). The calibers of the tumor vasculature were less than 2 mm in control and HIFU treated groups. The status of tumor microvessels destroyed in HIFU ablated group see Table 1, Figure 2C. Thinner microvessel caliber was associated with better ablated effect by HIFU. No destroyed changes was found in uterine tumor tissues with their supplying vessels in control group.

**Table 1:** Distribution of uterine tumor vascular calibers and HIFU ablation effect

Vascular diameter	Control group(n=15)	HIFU ablated group(n=15)	t value	p value
≤500μm	11.80±3.00	0	15.210	0.000
501~1000μm	7.27±1.83	0	15.371	0.000
1001~1500μm	1.07±0.35	0	11.879	0.000
1501~2000μm	0.59±0.24	0	9.404	0.000
>2001μm	0	0	0	0

#### Endotheliocyte UEA I detection

**Figure 3:**
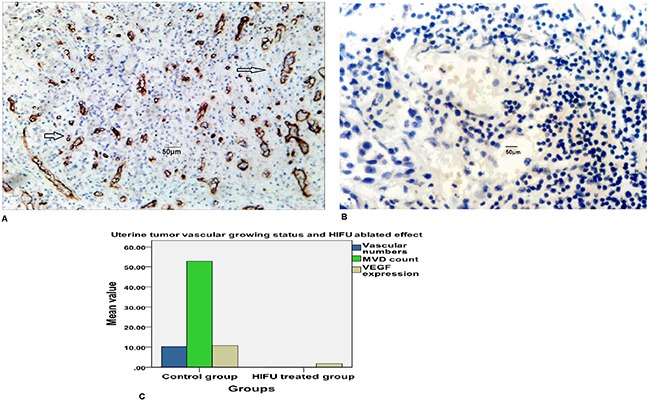
**A**. Microvascular UEA I label was deeply stained in control group (endothelial cell marked with arrows,400 times HPF). **B**. Microvascular UEA I label was not detected in control group (400 times HPF). **C**. Distribution of uterine tumor microvessels and HIFU ablated effect. The mean uterine tumor capillaries, venules and arterioles amounts together were 10.23 ± 2.35/vessels in control group, vascular structure was destroyed in HIFU ablated group, intact microvessels count was 0. UEA I staining was positive in control group, microvessel density count(MVD) was 52.73 ± 5.51/vessels/HPF, UEA I staining was negative HIFU ablated group, MVD count was 0. Vascular endothelial growth factor(VEGF) expression in control and HIFU ablated group were 10.77±2.32 versus 1.87±0.74 respectively, there was significant difference between two groups.

Microvascular UEA I label was deeply stained in control group. Microvessels appeared heterogeneous distribution within uterine cancer tissue. A vision with high microascular number was known as “hot spots”, it may be one endotheliocyte or one group of endotheliocytes (Figure 3A). The mean MVD number was 52.73 ± 5.51/vessels/HPF (400 times) in control group. Microvascular UEA I staining was not detected in HIFU treated group(Figure 3B), MVD count was 0. There was obvious different between control and HIFU treated group(t= 37.071 and P = 0.000). The mean amount detected of VEGF was 10.77±2.32 versus 1.87±0.74 in untreated group and HIFU treated group respectively, there was obvious different between two groups (t=14.969, p<0.000). Distribution of uterine tumor microvessels and HIFU ablated effect see Figure 3C.

#### Tumor vascular ultrastructure destructed by HIFU

**Figure 4:**
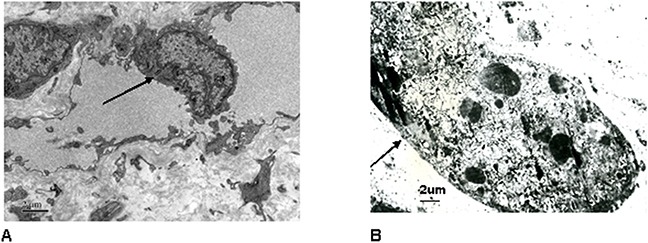
**A**. Endothelium cell ultrastructure of uterine tumor was not found injury in control group (endothelial cell marked with arrow, transmission electron microscope images, uranyl acetate and lead nitrate staining, 15000 times). **B**. The tumor microvascular ultrastructure was damaged in HIFU treated group. Endotheliocytes were dissolved. Stromal cells and cells surrounding vascular basilar membrane were disrupted(endothelial cell marked with arrow, transmission electron microscope images, uranyl acetate and lead nitrate staining, 6000 times).

Endothelium cell ultrastructure of uterine tumor was not found injury in untreated group (Figure 4A). The tumor microvessel ultrastructure was damaged in HIFU treated group. Endotheliocytes were dissolved. Stromal cells and cells surrounding vascular basilar membrane were disrupted (Figure 4B).

### X-ray DSA observation

X-ray DSA showed that defect architecture of uterine tumor microvessels included chaotic distribution, tumor staining and pooling of contrast material.

**Figure 5:**
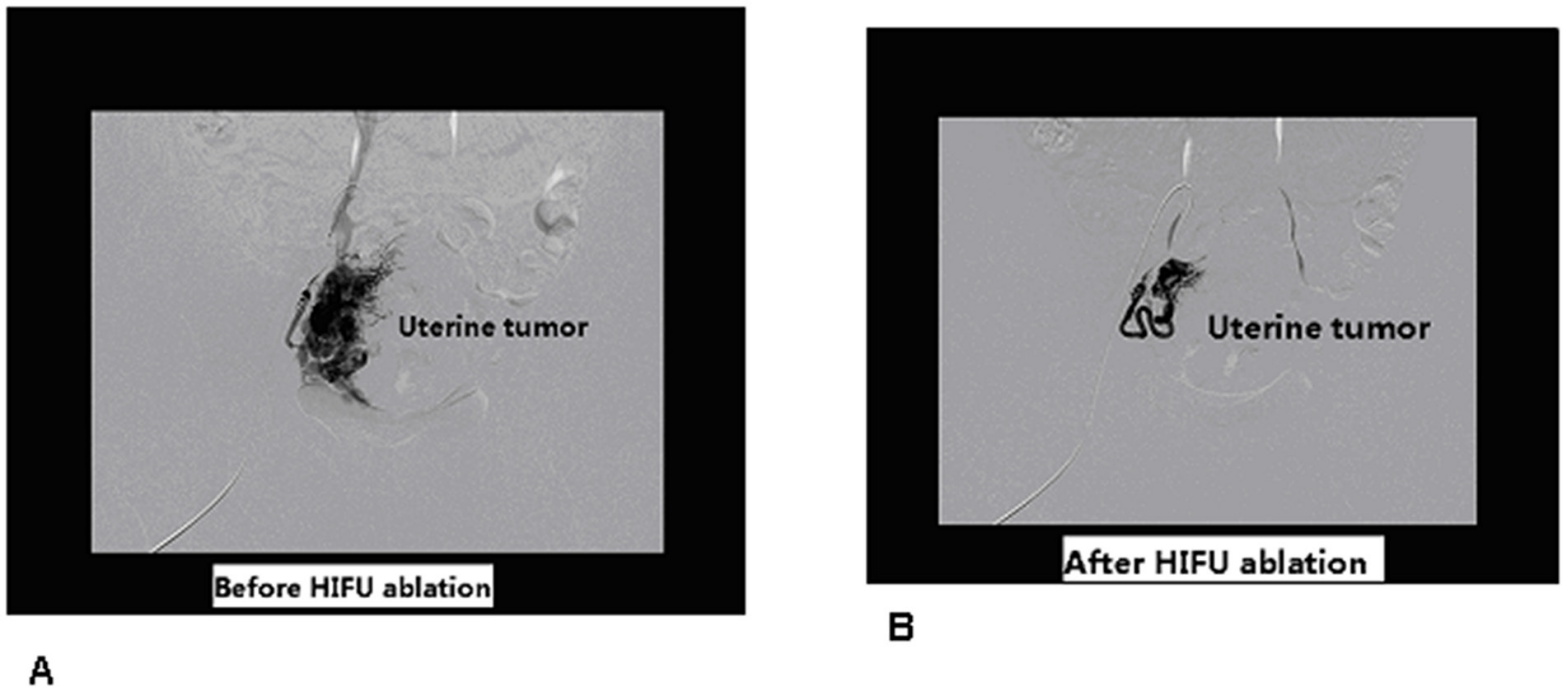
**A**. X-ray digital subtract angiography(DSA) of rabbit uterine tumor, before HIFU ablation, capillary phase presented typical tumor staining. **B**. After HIFU ablation, tumor vascular shadow were dismissed.

Endometrial carcinoma dynamic angiography could fully display the tumor feeding blood vessels of three phases, arterial phase, capillary phase, venous phase. DSA imaging allowed for visualization and measurements of vascular perfusion-related parameters, including whole process of contrast agent wash-in, parenchyma filling, and contrast agent wash-out. Arterial phase found tumor vascular dilation, distortion, displacement, irregular shape, and vascular lake filling. Branch artery may appear vascular straightening, displacement and stiffness. Capillary phase presented typical tumor staining(Figure 5A). Venous phase presented ‘veins appear early signs’ causing by arteriovenous shunt.

After HIFU ablation, uterine tumor vascular shadow were decreased or dismissed. (Figure 5B). The effective number of HIFU treatments in DSA group was as follows:excellent 7, efficacious 6, poor effect 2. Effective rate 86.7%(excellent 7+ efficacious 6,13/15), uterine tumor vascular shadow of DSA was decreased significantly than before HIFU ablation, p<0.001. see Table 2.

**Table 2:** Efficacy of HIFU ablation evaluated by tumor vascular functional imaging

Efficacy of HIFU ablation	Excellent number	Efficacious number	Poor effect number	*Effective rate*	X^2^	P value
X-ray DSA group after HIFU ablation	7(46.7%)	6(40%)	2(13.3%)	86.7%(13/15)	15.00	*p<0.001*
Ultrasonic CDFI group after HIFU ablation	7(46.7%)	7(46.7%)	1(6.7%)	93.3%(14/15)	15.00	*p<0.001*
Dye perfusion control group	15					
Dye perfusion HIFU ablation group	8(53.3%)	6(40%)	1(6.7%)	93.3%(14/15)	15.00	*p<0.001*

### Ultrasonic CDFI observation

**Figure 6:**
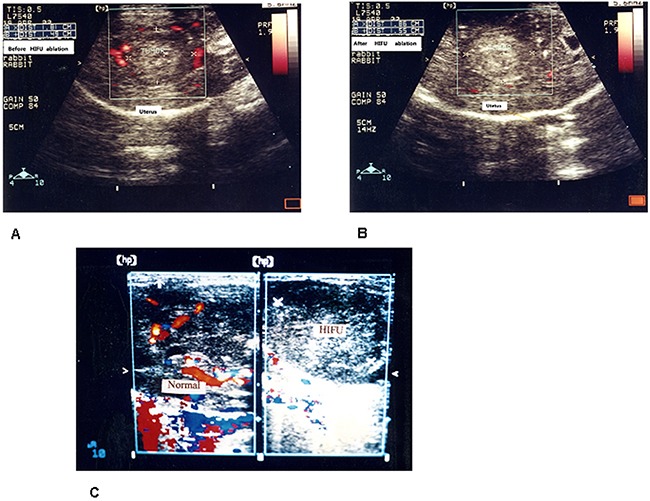
**A**. Ultrasonic color doppler flow imaging (CDFI) of rabbit uterine tumor, before HIFU ablation, tumor formed hypoecho image in ultrasonography, Ultrasonic CDFI revealed plenty of circuitous blood flow peripheral tumor nodules. **B**. After HIFU ablation, two-dimensional ultrasonography showed tumor nodules hyperecho change, tumors gray-scale enhanced. Ultrasonic CDFI showed tumor peripheral and internal blood flow signals were disappeared. **C. Left**: Ultrasonic CDFI, before HIFU ablation, rabbit tumor vessels presented dotted, linear or circuitous blood flow imaging. **Right**: After HIFU ablation, tumor gray-scale imaging enhanced, ultrasonic CDFI showed tumor peripheral and internal blood flow signals were disappeared.

Before HIFU treatment, urerine tumors appeared hypoecho imaging in ultrasonography, CDFI revealed tumor vascular blood flow signals peripheral and intratumoral tumor nodules. Most of tumor microvessels surrounded the tumor nodules and presented dotted, linear or cyclic blood flow signals. (Figure 6A, Figure 6C Left)

After HIFU ablation, ultrasonography showed tumor hyperecho change, tumor gray-scale enhanced. Ultrasonic CDFI showed tumor peripheral and internal blood flow signals were disappeared or significantly reduced(Figure 6B, Figure 6C Right)

In CDFI group, the effective number of HIFU treatments was as follows:excellent 7, efficacious 5, poor effect 1, effective rate 93.3% (excellent 7+ efficacious 5,14/15), tumor vascular blood flow signals of CDFI were decreased significantly than before HIFU ablation p<0.001. see Table 2.

### Dye perfusion for tumor vascular imaging

**Figure 7:**
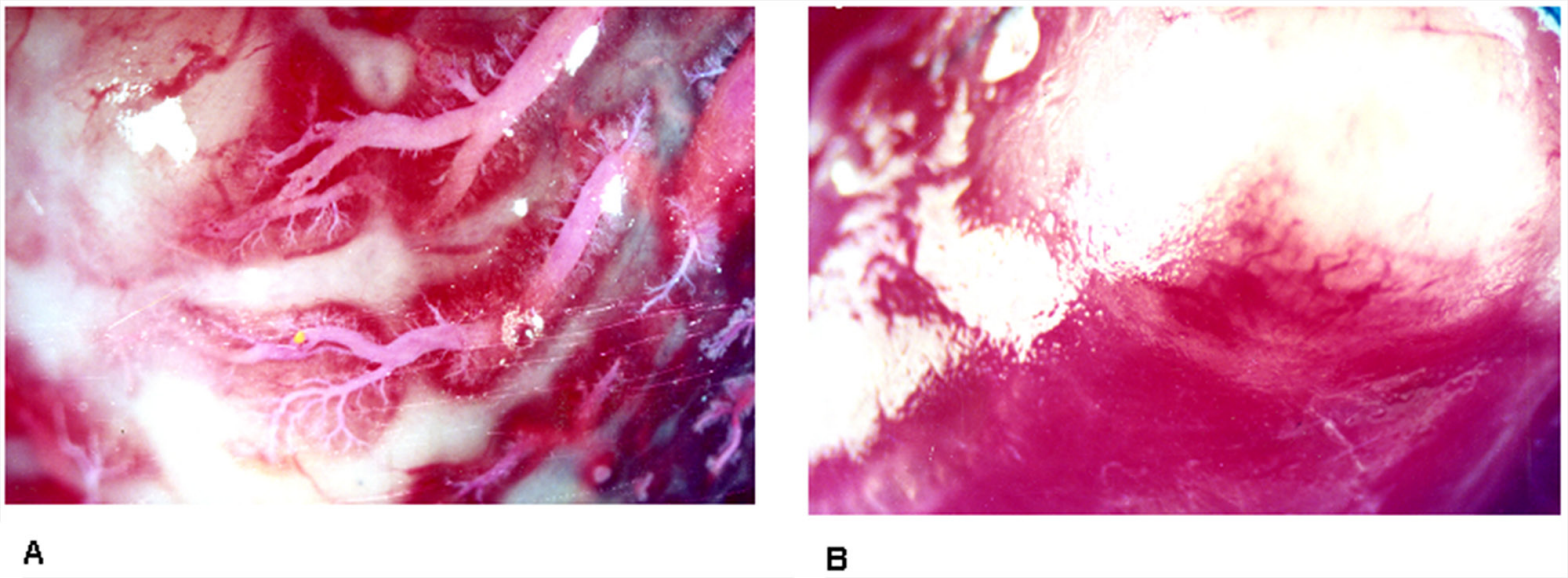
**A**. Intact microvessels of rabbit uterine tumor in control group were visualized by chloroprene latex dye perfusion at a magnification×10 objective lens. **B**. Microvesses of rabbit uterine tumor were not found filled with chloroprene latex dye at a magnification×10 objective lens.

Uterine tumor blood vessels were enlarged, distorted in irregular shape and filled with bright red dyes under anatomic microscope in control group(Figure 7A). Cross section showed rich blood supply was surrounded peripheral tumor nodules, relatively less blood supply was found intratumoral tumor nodules. Ischemia necrosis may be found in the central tumor due to without sufficient blood supply.

Dye perfusion performed after HIFU ablation, tumor vessels were not found filled with dye liquid(Figure 7B). HIFU ablation made tumor nodules become coagulative necrosis tissue blocks.

As showed Table 2, the effective number of HIFU treatments was as follows in dye perfusion group,:excellent 8, efficacious 6, poor effect 1. Effective rate was 93.7%(excellent8+ efficacious 6,14/15), p<0.001. The rates of tumor vascular dye perfusion in HIFU ablated group were reduced obviously than that of untreated group.

## DISCUSSION

### Endometrial carcinoma animal modality

Tumor animal modality is built to explore pathogenesis and treatment of human cancer. As a transplantable tumor lines, VX2 tumor can be passed on from generation to generation. As malignant tumor, VX2 tumor grows and spreads in different tissues of rabbits easily. Therefore implanted VX2 uterine tumor modality was adopted in this study. Overall process of uterine carcinoma progress and retroperitoneal lymph node invasion can be monitored [6–9].

Most endometrial carcinomas were adenocarcinoma, while rabbit VX2 tumor was squamous cancer. To our knowledge, endometrial carcinomas model usually adopted small animals such as mice, but mice were too small to convenient for performing tumor angiography and HIFU tumor ablation. The imaging was difficult discerned, for *in vivo* imaging in mouse need higher stereo and time distinguishing method than rabbits. Other large animals such as dogs had no endometrial adenocarcinoma model available.

Few uterine adenocarcinomas are found from animals, rabbit is one of them. This tumor lines can be implanted under the skin of rabbits as experimental disease modality. The tumor line may be cultivated within petri dish too. But there were no reports about whether this tumor tissue could be embedded to other rabbits. No further reports are available about this tumor line [10]. Upon to now there have been no endometrial adenocarcinomas line of rabbit available in the labouratory almost all over the world. According to reports in recent years, the rabbit endometrial carcinoma models are established still by VX2 tumor clones [6–9].

It was reported endometrial carcinoma model could be established by injection of tumor cells into rabbit endometrium [6–9]. But our experiment rabbit dissection revealed that injected tumor cells did not formed one tumor nodule, or not restricted within the uterine cavity, even implanted to peritoneum aimless, distant metastasized. Our *in vivo* uterine tumor model needed tumor cells form one solid nodule for convenient HIFU ablation. So in present study, an *in vivo* model of uterine tumor was established by embedded tumor blocks into the myometrium by surgical operation.

### Morphological and functional defect of tumor microvessels

Stimulated by vascular growing factors, tumor forms defect microvessels, lack of stromal cells support, sinus shape easily leaking vascular lumen. Tumor stem-like cells replace endothelium cells as part of vascular wall, this phenomenon called vascular mimicry. Microvessel density is higher in border of tumor than that of tumor center. Tumor vascular morphological defect cause chaotic blood stream, histanoxia in tumor tissues. Defect features of tumor microvessel cause tumor easily growth and spread, interfere with effects of chemical radiation treatment [3, 4].

### Effects of HIFU on tumor blood vessels

The energy of ultrasonic wave could be concentrated to make the focus region tumor tissue ablated, just like a magnifying glass could converge the ray of sun on one point. Using ultrasonic wave intensities between 5000 and 20000 W/cm^2^, one ultrasonic emission could make an objective tissue coagulative necrosis. The tissue coagulative necrosis could validated by ultrasonic imaging instantly find a strong echo range within the focus after each HIFU emission [11, 12].

HIFU destroy effect to vasculature is associated with microvessel caliber, blood stream speed and ultrasound features (rate of emission, intension and ablation time) [13].

A rat hepatic model was used detect HIFU ablated effect (4 MHz, 550 W/cm^2^,4 sec). The result confirmed HIFU could only destroy tumor microvascular caliber within 200μm. HIFU ablation destroyed tumor microcirculation and enhanced HIFU tumor treatment effect. A rabbit abdominal aorta model was used to detect HIFU treated effect (4 MHZ, 1,500 W/cm^2^, 5 sec), The experiment confirmed HIFU was security to large vasculature because of larger vascular caliber and higher speed of blood stream [14].

According to above experimental study and later literature reports, HIFU ablation effect on tumor blood vessels is correlated with temperature changes. when temperatures below 42°C, no vascular damage was found. Over 60°C, tumor capillaries were destroyed at once. In reality, the temperature of focal region reaches over 80°C during HIFU ablation, this induces tumor with their capillaries become necrosed tissues right away. There is a sharp temperature gradient between focus region and surrounding tissues, so no harm is induced by HIFU to adjacent tissues with their vessels [11, 12].

Directed by ultrasonic imaging precisely,60 rabbits with embedded uterine tumors were ablated by HIFU, no vascular rupture hematorrhea happened during the treatment. According pathological detection, tumor microvascular caliber were all within 2 mm. The results validated that HIFU treatment is safe to nearby larger vessels. HIFU monitoring by real time ultrasonic imaging can effectively destroy embedded endometrial tumor and tumor microcirculation without harming the surrounding tissues. Different types of tumor present resembling necrotic changes by HIFU ablation. The pathological changes confirmed that HIFU could thoroughly damage uterine tumor and their microvessels.

Tumor appeared cells shrinkage, nucleus deeply stained with HE detection after HIFU ablated. Cellular outlines were still distinct. Vascular composition was completely damaged. Vascular cellular intervals were indistinct, endothelium collapsed, cell nucleus disappeared, media vascular were disrupted. Thrombi were found within target vessels, Tumor vessels all become necrosed tissues. Necrotic tissue decomposition presented at 24 h after HIFU ablation.

The vascular elastan plates were decomposed in HIFU ablated group. Elastan layer arrangement was in chaos status. Furthermore, thinner microvessel caliber was associated with better ablated effect by HIFU. HIFU could thoroughly damage the uterine tumor microcirculation and larger vasculature with elastan. This could prevent early endometrial carcinoma cell from growth and dissemination. The effects of HIFU in tumor treatment were enhanced.

UEA I had been used as endothelial cell-specific markers for rabbit tumor endothelial cells. MVD count is a method validating tumor vascular growing condition. Endothelial cell staining was not detected in HIFU ablated group. This confirmed that proliferating microvessels had been thoroughly damaged. The degree of VEGF label reduced greatly in HIFU treated group than that of control group, the result proved tumor vascular growth was suppressed. According to observation, tumor microvascular ultrastructure was damaged, the result proved the effect of HIFU treatment again.

Shoji S et al, (2014) performed prostate carcinoma ablated by HIFU. Pathology found tumor microvessels degenerative change; endotheliocytes were separated from vascular wall. Endotheliocyte CD34 label was blurry or not detected. This clinical results are consistent with our above experimental results [15].

Vascular proliferation companied by tumor growth forms a tight connection. We performed different compositions of uterine tumor vascular structure detection. All of these tumor composition changes confirmed uterine tumors together with microvessels degenerative necrosis after HIFU ablation. As a microinvasive therapy, HIFU may break neovascularization correlated with tumor growth and spread tightly.

### Efficacy of HIFU evaluated by X-ray DSA

The rabbit uterine tumor imaging of DSA could offer an analogous change for human endometrial cancer. From above DSA imaging, functional perfusion and angiographic imaging was finished remarkably well in rabbit uterine tumor models. Advantage for this method was that it could be convenient for operation and economical. DSA imaging was used smoothly in above rabbit uterine tumor model as a functional imagery way to obtain blood perfusion by a mask subtraction method. The imaging obtained before urografin given was used to produce a mask, which was subtracted [16].

So rabbit uterine tumor model was adopted in above study because rabbit vascular diameter was large enough for DSA imaging observation and convenient for HIFU ablation. The treatment process was similar to clinical situation. DSA imaging could well determine tumor vascular caliber, winding degree, and microvessel density count. DSA imaging increased evaluation precision of HIFU ablated treatment [17].

In our above study, DSA showed that abnormal structure of tumor vascular chaotic distribution, tumor staining and pooling of contrast material. Dynamic DSA images fully displayed the uterine tumor of three phases, arterial phase, capillary phase, venous phase. After HIFU ablation, uterine tumor vascular shadow were decreased or dismissed. The result proved DSA could be exploited for successful *in vivo* preclinical uterine tumor vascular imaging, and DSA imaging showed that HIFU sonication could destroyed uterine tumor tissue and their vascularities.

### Efficacy of HIFU ablation evaluated by ultrasonic CDFI

The ultrasonic imaging offer real-time to visualize and measure the imbedded uterine tumor planar and spatial imaging in above rabbit model. Blood stream, vascular structure of tumor feeder vessels could be measured by ultrasonic CDFI. Tumor vascular perfusion and microcirculation could be detected by contrast agents *in vivo*. The latter was especially important in research of antiangiogenic treatment [18].

As microinvasive method, during tumor HIFU ablation process, ultrasonic imaging served as its self comparison in HIFU treatment, so it increased precision of the treatment and decreased the amount of experimental animals. Ultrasonoscopy can evaluate HIFU treatment effect follows-up in the same experimental rabbit.

Defect microvascular structure and blood flow are characters of tumor. It is important to detect these procedure in experimental study and clinical activity by noninvasive ways. In above study, ultrasonic CDFI could detect delicate changes in uterine tumor perfusion and microvasculature architecture. Ultrasonic CDFI detected blood stream imagine of uterine tumor vasculature before and after HIFU ablation when needed. The imaging of uterine tumor vasculature detected by ultrasonic CDFI was in agreement with microvessel density count of UEA I label, both methods confirmed HIFU ablation effect to uterine tumor vasculature [18].

Hiroyuki Fukuda et al., (2013) reported ultrasonic CDFI could be used to determine tumor position, distinguish hepatic internal structures in the middle of hepatic tumor treated by HIFU [19].

The uterine tumor HIFU scanning process is as above stated. HIFU ablation for uterine tumor was performed under ultrasound real-time monitoring. Before HIFU treatment, uterine tumor formed hypoecho imagine in ultrasonography, ultrasonic CDFI revealed plenty of circuitous blood flow echo peripheral and intratumoral tumor nodules. After HIFU ablation, ultrasonography showed tumor gray-scale enhanced. Ultrasonic CDFI showed tumor peripheral and internal blood flow signals were disappeared or significantly reduced.

### Efficacy of HIFU ablation evaluated by tumor vascular dye perfusion

A chloroprene latex dye for functional imaging of uterine tumor drainage was used in our HIFU sonication experiment. Bright red dye and chloroprene latex were mixed together. This mixture as a space-occupying contrast material was used to fill tumor microvessel. Chloroprene latex was a hydrophobic activation sol. This was a rapid and reliable method to produce tumor microvascular imaging in rabbit uterus.

Uterine tumor microvessels were filled and visualized with dye liquid. Dye liquid combined with endothelial cell membranes. The dye liquid labeled microvascular structures by perfusion. The specimens can be observed under anatomic microscope at low time vision. Serial optical sections can be acquired for spatial constructive imaging. This method can be used to observe whole body imaging of experimental rabbit or only uterine imaging [20].

In our dye perfusion control group, the tumor blood vessels showed enlarged, distorted in irregular shape and filled with bright red dye under anatomical microscope. In HIFU treatment group, HIFU ablation made tumor nodules become coagulative necrosis tissue and blocked tumor blood perfusion. Dye perfusion performed after HIFU ablation, tumor vessels were not found filled with dye perfusion.

### Antiangiogenic methods versus HIFU treatment

Compared too many types of tumor cells, genetic types of tumor vasculature was simple and stable, so tumor microvessels replaced tumor cells as a target. The treatment resistant may be less encountered. This was the original purpose of antiangiogenic therapy. The whole course of tumor vasculogenesis is far from complex than first expected. Although experimental observation and clinical trials confirmed antiangiogenic therapy presented some antitumor effects, the effects were dissatisfied. Resistant to the antiangiogenic agents and side effects were still found [21].

Thalidomide is an only antiangiogenic drug that is used in endometrial cancer, but thalidomide showed only 25% clinical benefit rate among patients with recurrent endometrial cancer [1].

Zongwei Wang et al, (2015) presented ten respects of tumor vascularization that may be used as targets in anti-angiogenic treatment including architectural deformity of tumor vasculature and so on. This ten respects of tumor vascularization confirmed its complicated. He recommended ten native compounds that may be used as anti-angiogenicagents. The combination of these agents may reduce side effects and resistant to the therapy, increase treatment effects. But these natural compounds must be changed into absorbable nanoparticles before they can be used in clinical treatment. So this is just an envisage [3].

The relationship of tumor microvessels and tumor cells is that of tumor cells and their stromal components. Tumor stroma play central role in tumor growth and spread. Vascular proliferation companied by tumor growth forms a tight connection. To cure cancers, cancer tissues with their microcirculation must be destroyed together [4, 22].

We first performed histological examination as fundamental evaluation for effects of HIFU ablation in anti-angiogenesis uterine tumor models. All of tumor composition detected validated uterine tumor tissues and their microvessels coagulative necrosis after HIFU ablation.

Next, blood flow imaging was used to evaluation of HIFU ablation effects. X-ray DSA visualized tumor capillaries, determined tumor vascular caliber, microvessel density. Tumor blood flow quantified by ultrasonic CDFI. Dye perfusion simplified the visualization of tumor blood vessels and data analysis. So vascular imaging provided functional and targeted information for HIFU treatment. The imaging changes confirmed uterine tumor vascular perfusion had been blocked by HIFU ablation.

This results indicated HIFU ablation could make uterine tumors and their microvessels become necrosed tissues. This may block tight connection of vascularization and uterine cells growth, and get ride of uterine carcinoma.

### Development of uterine HIFU treatment

HIFU could cause uterine tumors and their microvessels become necrosed tissues through the intact skin of experimental rabbits. The results confirmed HIFU was a perfect therapy that could make uterine carcinomas along with their microvessels destructed.

The advantages of HIFU treatment were as following: without hemorrhage, no radiation, repeatable, minimal invasive, preserving the structure and reproductive function of uterus, no tumor resistance, microinvasive [11, 12].

HIFU maybe used on endometrial carcinoma clinical treatment. The treatment process must depend on imaging system guidance. This included ultrasound or magnetic resonance imaging(MRI). Ultrasound is cheap, can be used easily and widely, transmits lively HIFU ablation imaging. Detection of MRI is sensitive and can measure tissue temperature. Combination of two methods may increase effective guidance during uterine tumor HIFU ablation process [5, 11, 12].

This experiment validated, with ultrasound guidance, uterine tumor was ablated smoothly by HIFU. Ultrasonic imaging could determine uterine tumor position precisely in the middle of process of uterine carcinomas HIFU ablated.

Tumor vascularization determines tumor growth and spread. Now ultrasound can offer tumor blood flow functional imaging. With tumor vascular molecular imaging, targeted therapy may further increase effect of uterine tumor during HIFU ablation in future [22].

## CONCLUSIONS

We chose embedded uterine tumor animal modal, adopted pathologic and functional imaging methods to determine effectiveness of HIFU on early stage endometrial carcinoma. The results validated uterine tumors together with microvessels become necrosed tissues by HIFU. This may block tight connection of vascularization and uterine carcinoma development. As a minimally invasive modality, HIFU ablation can not cause tumor resistance. HIFU maybe an alternative approach of targeted therapy and new antiangiogenic strategy for endometrial cancer.

### Strengths and limitations of this study

- Rabbit endometrial carcinoma models were established via tumor implanting methods. A randomized control study was performed.
- Evaluation of the effect of HIFU ablation to targeted tumor vascular structure by HE staining, fiber staining, endothelial cell staining. Ultrastructure of vasculature was observed by electron microscopy. Pathologic changes confirmed tumor vascular structure components including elastic fiber, endothelial cells all were destroyed by HIFU ablation.
- Assessment of the effect of HIFU ablation to targeted vascular function by X-ray DSA, ultrasonic CDFI, chloroprene latex dye perfusion. The imaging changes confirmed uterine tumor vascular perfusion had been blocked by HIFU ablation.
- Pathologic and imaging changes validated that HIFU could whole destroy endometrial tumor cells and their vascularities.
- This study only included embedded single nodule of early stage endometrial carcinoma, not included multifocal endometrial carcinoma nodules or advanced endometrial carcinoma. No long time following up was carried out after HIFU treatment.

## MATERIALS AND METHODS

Experimental animals: 92 female experiment rabbits included experimental rabbits 90 and donor rabbits 2, 3~4 months old, weight 2~3 kg were supplied by Zhabei district central hospital animal center. Rabbit implanted endometrial carcinoma models were established via implanting VX2 tumor blocks into uterine muscularis mucosae, Contents of experiment included:

(1) Pathologic group, experimental rabbits were divided into 2 subgroups, Control group performed pathologic observation via autopsy directly, HIFU treatment group performed pathologic observation after HIFU treatment(Pathologic group, n=30).

(2) Digital subtract angiography(DSA) group, uterine X-ray DSA was performed before and after HIFU ablation(DSA group, n=15).

(3) Color Doppler flow imaging(CDFI) group, uterine CDFI was performed before and after HIFU ablation. (CDFI group, n=15).

(4) Dye perfusion group was divided into 2 subgroups, Control group performed chloroprene latex dye perfusion directly, HIFU treatment group performed chloroprene latex dye infusion after HIFU ablation. (Dye perfusion group, n=30).

This research was allowed by the ethics committee of Zhabei district central hospital, an affiliated hospital of second military medical university.

All animals received humane care in compliance with the principles of laboratory animal care formulated by the national society of medical research and the guide for the care and use of laboratory animals, published by the US national institutes of health. The protocol was approved by the animal care and use committee of Zhabei district central hospital. Experimental trial registration number:2015-036.

Rabbits VX2 squamous cancer cells were purchased from Funabashi company from Japan.

### Rabbit VX2 endometrial carcinoma development and propagation

Development and propagation of the VX2 cell line were described previously [23].

### VX2 endometrial carcinoma implantation

The abdominal skin of experiment animals were shaved with 8% N_2_S. The tumor blocks were embedded in sterile surroundings. The rabbits were anaesthetized by 3% pentobarbital sodium at dose 30 mg/kg.

A vertical incision was made on the lower abdominal midline. After the experimental animal uterus was exposed, one incision about 0.5 cm was made by ophthalmic scissors at the uterine abdomen side, the incision was deep into muscularis mucosae and 1 cm away from the cervix, two 1~3 mm tumor blocks were implanted into the uterine incision. The incisions of uterus and abdomen were closed by absorbable sutures separately.

After the operation, the rabbits were aroused and sent back to cages. The rabbits received 800,000 U penicillin injection per day, sterilized incision within 3 days, and monitored wound healing and appetite.

### Tumor growth monitoring

Experiment animals survival status and tumor volume were followed up by ultrasonic and autopsy of general observation. Autopsy was taken from uterus, local lymph nodes, and other tissues that tumor may spread.

Tumor sizes were detected by vernier caliper. The tumor volume(V) was counted according to “V=A×B^2^/2”(A stood for maximum diameter, B presented transverse diameter).

After tumor embedded one week later, ultrasound was used to detect uterine tumor growing status (tumor volume, nodule number).

After tumor implanted 3 weeks, when maximum tumor diameter was about 1.5cm, tumor nodule only within myometrium, experimental animals received HIFU ablation.

**Figure 8:**
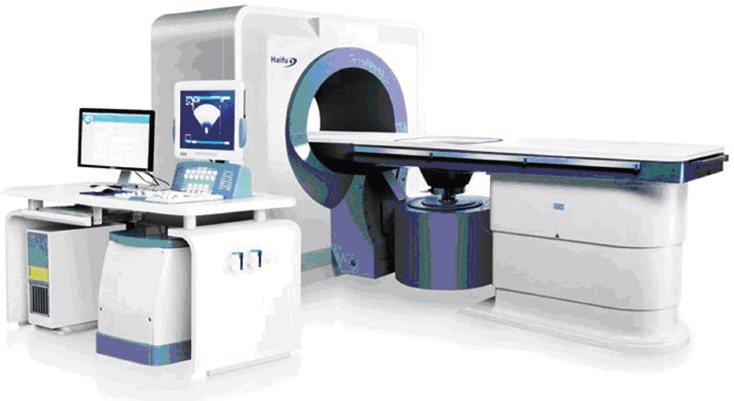
The high intensity ultrasonic JC tumor treatment system

*Treatment device and its component*s: the high intensity ultrasonic JC tumor treatment system was produced by Haifu Medical Technology co., LTD, Chongqing, China(Figure 8). The HIFU therapeutic system and its parameters used were described previously [24]. Briefly, the system was mainly consisted by a diagnostic ultrasound device, a treatment ultrasound device and an automatic computer control unit.

A monitoring imaging ultrasound probe was acted as realtime guidance during the HIFU treatment procedure. The ultrasonic beam produced by treatment transducer and image of the monitoring probe thoroughly interweaved. The combining transducer driving by electric motors could be moved in three-dimensional space exactly. The treatment was precisely controlled by computer system.

The course of HIFU ablation: The abdominal skin of experiment animals were shaved with 8% N_2_S again if necessary. After general anesthetized by pentobarbital sodium, the experimental rabbit was fixed in prone position with the abdominal skin immersed into de-aerated water on treatment bed. The ultrasonic beam crossed the skin and focused on uterine tumor tissue. Diagnostic ultrasound imaging was used as a contrast, HIFU beam focus located in the treatment range. The HIFU treatment system incessantly ablated the target tissue. The scope included tumor and 2 cm of uterine tissue surrounding the tumor. Ultrasonic imaging acquired after HIFU ablation was compared to that acquired before HIFU ablation as treatment reference. After HIFU treatment, it was confirmed excellent effect when strong echo region presented and blood flow vanished in uterine tumor monitoring ultrasonography.

With diagnostic ultrasound guidance, the HIFU continuous scanned layer by layer of uterine tumors till tumors were thoroughly ablated. In this study, the sweep power was adopted from 100W to 400W at last. Every scanning time was 5 sec. The transducer moved on 20 mm long track with sweeping speed 1–3mm/sec. HIFU ablation time ranged from 20 to 45 min(median, 35 min) per experimental rabbit.

### Tumor pathological observation

The tumor tissues were fixed in 10% neutral formaldehyde solution, embedded in paraffin, sectioned, placed on slides. One slide was stained with hematoxylin and eosin (HE), one slide was used for elastic fibers staining, one slide was used for endothelial cell staining and one slide for endothelial cell growth factor(VEGF) staining. All slides were observed under an optical microscope.

#### Elastic fiber staining

The agent preparation way for Victoria blue and Ponceau's staining, the process of elastic fiber staining were described previously [24].

To determine elastic fiber destroying status, a microscopic micrometer was used to detect tumor vascular diameter. A microscopic micrometer was constituted by eyepiece micrometer and stage graticules, both of them must be used together. According to measurement, one eyepiece micrometer stood for 4.8 μm at 100 times magnifications, and one eyepiece micrometer stood for 1.2 μm at 400 time magnifications. Blood vessels were searched at 40 times magnification and then destroyed vascular were determined at 400 time magnification. For each specimen, 5 visions were added, and the mean vascular caliber of HIFU ablation was counted.

#### Ultrastructural observation

Tumor tissues were divided into 1 mm^3^ sections. Half of the section was produced by the CQR-1 type ultra-microtome technique, and ultrathin sections were produced after location. Uranyl acetate stained the sections first, then lead nitrate stained the sections successively. The sections were taken picture by H-600 transmission electron microscopy.

#### Tumor-derived vascular endothelial UEA I staining

The avidin-biotin peroxidase complex (ABC) technique was used. Ulex europaeus agglutinin(UEA) I content was 20 μg / ml. UEA could be bonded with fucose of endothelial cells, then UEA were labeled using DAB(diaminobenzidine) substrate.

Microvessel density(MVD) count:a single endothelial cell and a cell group positively stained with UEA was regarded as a microvessel. MVD count was not according to visible separate vascular lumina. Blood vessels that had muscle layer visible or a vascular more than eight red blood cells in diameter were examined by HE staining. Elastic fibers were stained and observed separately. The process of MVD count was according to our previously report [24].

#### Evaluation of VEGF expression

VEGF staining by immunohistochemistry and the standard of scores semiquantitatively were described previously [24].

### X-ray DSA observation

After endometrial tumor implanted 3 weeks, tumors grew to approximately 1.5 cm in diameter, the rabbits were used for the experiment. The abdominal skin of animals were shaved with 8% N_2_S. Fasted 6 h before the experiment, animals were anesthetized with 3% pentobarbital sodium at dose 30 mg/kg via the ear vein and then placed in a supine position. X-ray DSA was used C-arm unit (Digitex a, shimadzu, Japan). After sterilization, using the Seldinger technique, 4-F cobra-shaped introducer sheath (Cordis Corp., Miami Lakes, FL, USA) was inserted into the right-sight femoral artery by a guide wire and assured with a 1-0 silk suture. A 3-F catheter was introduced by the guidewire through the sheath and advanced to right internal iliac artery. Angiography of the common iliac bifurcation and the internal iliac artery and its branches was obtained with 30-35% urografin. The catheter and guidewire were then used to superselect to the right uterine artery. After the tip of the catheter had been placed into the uterine artery, DSA was performed using 3-4 ml of contrast agent 30-35% urografin at an inflow rate of 0.8-1.2 ml/s.

Dynamic DSA images of each uterus of experimental were reviewed. The early phase images were used to determine the feeding arteries of the tumor. The scores of such arteries were evaluated according to their contributions to the tumor stain in the later phase images. The accumulative scores of each uterine artery of 15 experimental animals were used to evaluate the blood supply of feeding arteries to the tumors.

In X-ray DSA group, a scoring method was used to estimate for HIFU ablation efficacy according to tumor vascular perfusion to the tumor stain in dynamic DSA images. More than 75% tumor stain disappeared was scored as excellent, 25%-75% as efficacious, < 25% or tumor stain did not changed as poor efficacy.

### Ultrasonic CDFI observation

After endometrial tumor implanted 3 weeks, The abdominal skin of experiment animals were prepared as above described. CDFI was used ultrasonic device of AU3 ultrasound imaging device (Esaote, Genoa, Italy) at the frequency of 3.5 MHz. An ultrasonic examination was performed before and after HIFU treatment respectively. Tumor mass was detected with two-dimensional ultrasound at the abdominal original incision site. Tumor gray scale was observed first, CDFI was detected further.

In CDFI group, a scoring method was used to estimate for HIFU ablation efficacy according to tumor vascular CDFI signals. More than 75% blood flow perfusion signals disappeared were scored as excellent, 25%-75% as efficacious, < 25% or tumor blood flow signals did not changed as poorefficacy.

### Chloroprene latex dye perfusion for tumor vascular imaging

#### Chloroprene latex dye preparation

20 g bright red dyes were dissolved in 30 ml of detergent. Both of them were stirred well. Then the above liquid was mixed with 300 ml chloroprene latex. This chloroprene latex dye mixed liquid can be used for tumor vascular perfusion.

#### Chloroprene latex dye tumor vascular perfusion

Animals in control group performed chloroprene latex dye perfusion directly, animals in HIFU treatment group performed chloroprene latex dye infusion after HIFU ablation.

Rabbits fasted 6 h before the experiment. Experiment animals in both groups were anesthetized with 3% pentobarbital sodium at dose 30 mg/kg via the ear vein and then placed in a supine position. The former operating scar incision was made on the lower abdomen. Bladder and bowels were covered with gauze. After blunt separation, the abdominal aorta was exposed, secured with a 1-0 silk suture at distal abdominal aorta near iliac artery bifurcation.

The chloroprene latex dye was sucked into 20 ml syringe connected 16-gauge needle.

After the needle punctured into the abdominal aorta successful, the dye liquid was injected into blood vessels slowly. Uterine blood sinus and tumor capillaries of animals were gradually filled. After tumor vascularities had been filled by dye liquid completely, the infusion was stopped. The experimental uteruses were harvested, observed and photographed by anatomic microscope.

In the dye perfusion group, a scoring method was used to estimate for HIFU ablation efficacy according to tumor vascular dye perfusion status. More than 75% vascular dye filled disappeared was scored as excellent, 25%-75% dye filled disappeared as efficacious, less than 25% or tumor dye perfusion did not changed as poor efficacy.

### Statistical analysis

The data in the present study were showed as mean±SD, t-student test and correlation analysis were used to analyze the variance in different groups. Chi-square test were used to analyze the variance in different groups for efficacy rate of HIFU ablation(%). SPSS 19.0 software (SPSS, Inc.) was used for the statistical analyses of the data. It was considered there was significant differences when p was less than 0.05.

All experimental animals were general anesthesia by 3% pentobarbital sodium at dose 30 mg/kg during HIFU ablation and other intervention methods. When experiment have been finished, all experimental animals were sacrificed by over dose of 3% pentobarbital sodium. No experimental animals died as a result of the intervention before the end of the study. No long time follow up for experimental rabbits survive rates was carried out after HIFU ablation in all experimental rabbits.

### Ethical approval

All applicable international guidelines for the care and use of animals were followed. The study was approved by the ethics committee of Zhabei district central hospital. Experimental trial registration number:2015-036.

### Data sharing statement

No additional data are available.
